# Relationships between physical properties and sequence in silkworm silks

**DOI:** 10.1038/srep27573

**Published:** 2016-06-09

**Authors:** Ali D. Malay, Ryota Sato, Kenjiro Yazawa, Hiroe Watanabe, Nao Ifuku, Hiroyasu Masunaga, Takaaki Hikima, Juan Guan, Biman B. Mandal, Siriporn Damrongsakkul, Keiji Numata

**Affiliations:** 1 Enzyme Research Team, RIKEN Center for Sustainable Resource Science, 2-1 Hirosawa, Wako-shi, Saitama 351-0198, Japan; 2 Japan Synchrotron Radiation Research Institute, 1-1-1, Kouto, Sayo-cho, Sayo-gun, Hyogo 679-5198, Japan; 3 RIKEN SPring-8 Center, 1-1-1 Kouto, Sayo-cho, Sayo-gun, Hyogo 679-5198, Japan; 4 School of Materials Science and Engineering, Beihang University, 37 Xueyuan Road, Beijing, 100191, China.; 5 Biomaterial and Tissue Engineering Laboratory, Department of Biosciences and Bioengineering, Indian Institute of Technology Guwahati (IITG), Guwahati, 781039, India; 6 Department of Chemical Engineering, Faculty of Engineering, Chulalongkorn University, Bangkok, 10330, Thailand

## Abstract

Silk has attracted widespread attention due to its superlative material properties and promising applications. However, the determinants behind the variations in material properties among different types of silk are not well understood. We analysed the physical properties of silk samples from a variety of silkmoth cocoons, including domesticated *Bombyx mori* varieties and several species from Saturniidae. Tensile deformation tests, thermal analyses, and investigations on crystalline structure and orientation of the fibres were performed. The results showed that saturniid silks produce more highly-defined structural transitions compared to *B. mori*, as seen in the yielding and strain hardening events during tensile deformation and in the changes observed during thermal analyses. These observations were analysed in terms of the constituent fibroin sequences, which in *B. mori* are predicted to produce heterogeneous structures, whereas the strictly modular repeats of the saturniid sequences are hypothesized to produce structures that respond in a concerted manner. Within saturniid fibroins, thermal stability was found to correlate with the abundance of poly-alanine residues, whereas differences in fibre extensibility can be related to varying ratios of GG*X* motifs versus bulky hydrophobic residues in the amorphous phase.

Silkmoth larvae construct cocoons out of silk fibres as a protective strategy against infection, physical injury, and changes in temperature and humidity during the vulnerable stage of pupation^1^,^2^,^3^,^4^, and humans have utilized silk for millennia to produce textiles of great value and beauty. Recently, silk has also become a major area of study in the fields of materials science and biomedical engineering due to its remarkable strength, toughness, biocompatibility and biodegradability^5^. There have been numerous reports on the synthesis of silk-inspired materials, such as fibres, hydrogels and films, aimed toward the development of various applications that include tissue regeneration and sustained-release drug delivery systems^6^,^7^,^8^. Adding interest are studies showing that silk fibres obtained from different species of silkmoth possess different material properties^9^,^10^,^11^,^12^. However, the determinants of these differences are not clear, and thus understanding of their origins is highly desirable.

Silkworm silk is a protein fibre composite comprising a semi-crystalline fibroin core, mainly responsible for the load-bearing capacity, and an outer layer of sericin, which functions as a gumming agent^13^. Fibroin heavy chain are large structural proteins with an overall architecture consisting of two non-repetitive terminal domains and a long, internally repetitive central domain, corresponding to >90% of the total length of the coding sequence, and made up of multiple concatenated amino acid motifs^14^,^15^. The repetitive sequences of fibroins have undergone extensive sequence diversification among the different taxa, as a consequence of evolutionary mechanisms that operate on DNA with long, highly repetitive sequences^16^,^17^,^18^.

There has been much discussion regarding the extent to which the fibroin amino acid sequences affect the material properties of different silk types. On the one hand, there is clearly a relationship between certain sequence motifs and the ability of the fibroin polypeptides to adopt the typical structure consisting of stacked β-sheets and amorphous regions characteristic of the semi-crystalline silk polymer^19^,^20^. Both GAGAGS and AAAAAA motifs can adopt stacked β-sheet conformations to constitute the main crystalline component of the fibers, which lead to predictable changes in material properties, such as differences in thermal stability and the packing of the β-sheets^21^,^22^. Certain combinations of amino acid motifs are thought to go hand-in-hand with specialised biological functions^23^. Furthermore, the genes encoding fibroins are clearly under selective pressure to preserve specific sequence features, as seen in the fact that certain combinations of amino acid motifs appear again and again in the sequences of silk proteins from divergent taxa^24^. On the other hand, ample empirical evidence has shown that extrinsic parameters can also have a major impact on the material properties of silk fibres^25^,^26^. Further investigations are clearly needed to resolve this issue.

We present a broad comparative survey on silkworm silks with two main aims. The first aim is to investigate various material properties of a wide array of lepidopteran silk types found across Asia, in terms of mechanical properties, thermal characteristics, and crystalline structure. Secondly, we address the question regarding the influence of the polypeptide primary structure on silk material properties by analysing the experimental results in terms of the underlying fibroin amino acid sequences from the different species.

Fourteen types of cocoon silks were investigated, corresponding to eight species of silk moths. Included are four varieties of silk from the domesticated silkmoth, *Bombyx mori*, and several species belonging to the family Saturniidae, including *Antheraea* and *Samia* silks that produce high-quality textiles (such as tussah, tensan, muga, and eri silk varieties), and several wild silk moths, *Actias aliena*, *Rhodinia fugax*, and *Saturnia jonasii*, whose properties are still largely unknown. To minimize effects of inter-individual variations and differences arising from varying rearing environments, samples were collected from different geographical locations whenever possible. We used native silk fibres unravelled directly from the cocoons, rather than degummed fibres that have been subjected to harsh chemical treatments, to minimize changes in properties that result from such methods^27^,^28^.

## Results

### Morphology

**Figure 1:**
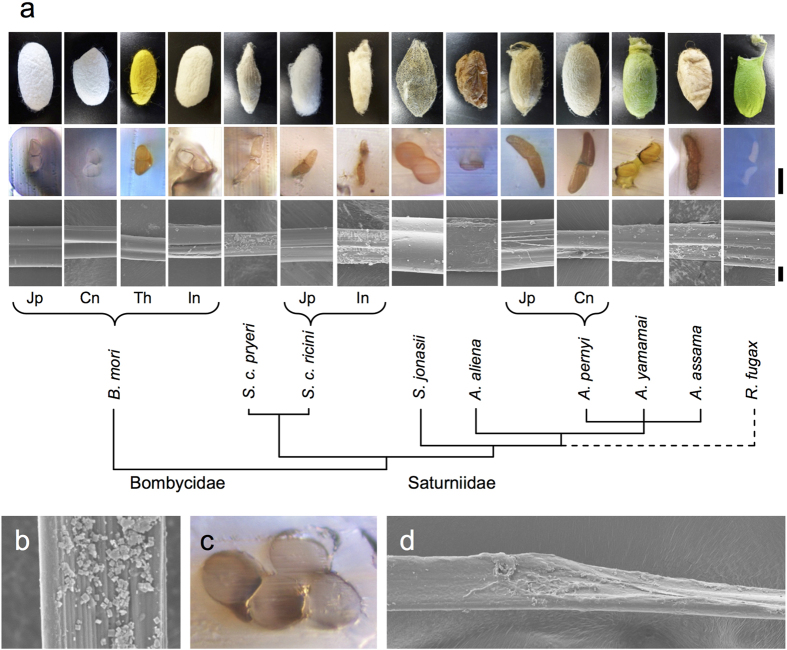
Morphologies of the different silks used in this study. (**a**) *First row*, representative photographs of cocoons (not to scale); *second row*, representative photomicrographs showing cross-sections of silk fibres embedded in cyanoacrylate glue (scale bar = 20 μm); *third row*, representative SEM images of fibre surfaces (scale bar = 10 μm). The phylogenetic assignment is based on Sima *et al*.^47^. Branch lengths are arbitrary, and the position of *R. fugax* is tentatively assigned. (**b**) SEM image showing the presence of calcium oxalate crystals on the surface of *S. c. pryeri* silk fibre. (**c**) Cross-sectional photomicrograph showing the common occurrence of silk fibres stuck together with gum material in *S. jonasii* cocoons. (**d**) SEM image showing an example of abrupt changes in the morphology and fibre diameter in *A. aliena*.

The morphological features of the silks investigated in this study are presented in Fig. 1a. Variations in cocoon appearance, fibre packing density, degree of bonding, and cross-sectional shape were observed. The *B. mori* cocoons exhibited a high degree of porosity and relatively loose bonding with sericin gum^29^, which correlates with the relative ease of unraveling the fibres from the cocoons. *Antheraea* cocoons featured thicker fibres that were densely packed, whereas *S. c. ricini* generated more loosely packed fibres. In *A. aliena* cocoons, the low porosity and brittle consistency of the silk made unraveling silk of extended fibre lengths difficult, whereas in *R. fugax*, fibres were densely packed and almost uninterruptedly coated with sericin. The cocoon from *S. jonasii* featured a lattice-like morphology with large apertures (>1 mm diameter), with fibres frequently occurring in thick bundles heavily bonded with sericin.

Light microscopy of cross-sections and SEM surface visualization showed details of the fibre dimensions and morphology (Fig. 1a). In all cases, the native fibres (baves) consisted of two filaments or brins surrounded by an outer layer of sericin. The *B. mori* silk samples typically displayed triangular cross-sections, with considerable variations in surface areas. SEM images revealed a mostly intact sericin layer surrounding the fibres, occasionally with thin cracks or locally abraded regions (not shown). In *Antheraea*, *Samia*, and *R. fugax*, the silk fibres had flattened or wing-shaped cross-sections, although variations were also common (*e.g.*, as shown for *A. yamamai*). Under SEM, cubic crystals were frequently observed on the surface of the saturniid silks (shown in Fig. 1b for *S. c. pryeri*, with crystal dimensions of 0.5–2 μm on a side), corresponding to the secretion of calcium oxalate by the larvae during cocoon construction^29^. Overall, the *A. aliena* fibres showed the greatest structural heterogeneity, with frequent splitting and drastic changes in fibre diameter and morphology (Fig. 1c), which may help account for their poor mechanical properties (see below). In *S. jonasii*, the fibres were especially rounded and thick (typically >20 μm in diameter) and were typically encountered in bundles that were firmly bonded together (Fig. 1d).

### Mechanical properties

The results of tensile deformation tests, which measured the ultimate tensile strength, extensibility (elongation to break), Young’s modulus, and toughness (energy to break) of individual silk fibres are summarized in Table S1; representative stress-strain curves of each of the samples are shown in Fig. S81. The overall results fall within the range of expected values, although with a large data spread and a generally low level of reproducibility even between fibres from the same cocoon, as previously noted^9^,^10^,^27^,^30^,^31^. Certain trends were noted, *e.g.* the higher initial elastic modulus observed for Indian *B. mori* silk (with a mean value of 8.6 GPa) compared to the other *B. mori* samples, which may reflect differences in the local rearing environments. However, taken as a whole, no obvious correlations could be made between phylogeny and either the tensile strength or elastic modulus of the different samples, with mean values falling between 0.34–0.57 GPa and 4–8.6 GPa, respectively. On the other hand, the samples could be classified into two groups according to extensibility: those exhibiting higher strain to break included *B. mori*, *Antheraea*, and *Samia* silks, with an overall mean range of 23–36%. The remainder of the samples, namely *A. aliena*, *R. fugax*, and *S. jonasii*, displayed inferior extensibility values, ranging on average from 14–22%. As a consequence of the differences in extensibility, the calculated toughness values were also divergent between the two groups, which ranged from 0.06–0.1 GJ m^−3^ for the high extensibility silks and 0.03–0.06 GJ m^−3^ for the low extensibility silks.

**Figure 2:**
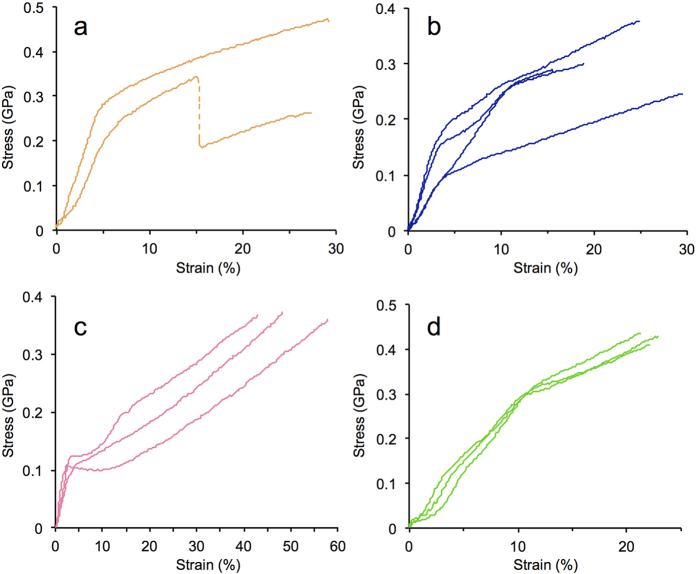
Stress-strain analyses of native silk fibres. Representative results are shown for (**a**) *B. mori* Jp, (**b**) *A. pernyi* Jp, (**c**) *S. c. ricini* Jp, (**d**) *R. fugax*.

Analysis of the tensile deformation curves showed that the different silk types followed different stress-strain paths, with qualitative features correlating with phylogeny (Fig. 2). Typical results of the *B. mori* tests are shown in Fig. 2a, which exhibited either simultaneous or separate breaking of the two strands upon the extension of force. The overall tensile path can be described as an initial elastic region of high modulus followed by an indistinct yielding region at approximately 5 to 10% strain and leading to a region with a nonlinearly decreasing slope toward eventual failure, consistent with previous reports^13^,^32^. Saturniid silks, in contrast, displayed stress-strain paths with generally more well-defined transitions. Figure 2b shows typical deformation curves for *A. pernyi*, representative of other *Antheraea* samples, which feature an initial elastic region and a prominent yield point at approximately 4% strain, followed by a post-yield region with significant differences in modulus among individual samples and frequently exhibiting sigmoidal strain hardening. A second yield point was typically observed at approximately 10% strain, followed by a region of lower slope until tensile failure^10^,^11^,^33^. *Samia* silk produced somewhat similar yet distinctive tensile behaviours, with a prominent yield point at around 4% strain usually followed by a plateau region that leads to an extended, uninterrupted strain-hardening region, until failure (Fig. 2c). The silk fibres from *R. fugax* featured two well-defined yield points at approximately 4 and 10% strain, although the extensibility was relatively low, with average breaking strains at approximately 20% elongation (Fig. 2d).

### Thermal analyses

**Figure 3:**
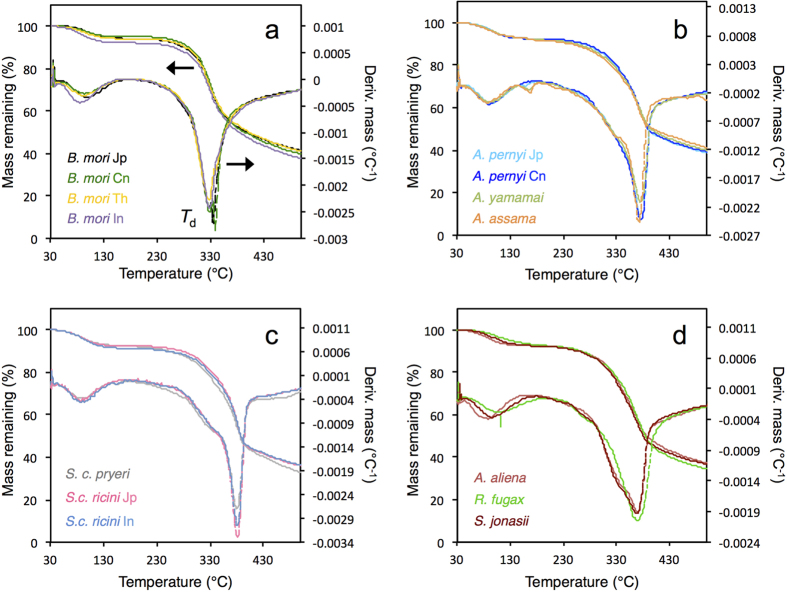
Thermogravimetric analyses of the different cocoon samples. Each panel combines the percent mass loss data (TG: *top curve, left axis*) and the first derivative plots of the percent mass remaining (DTG: *bottom curve, right axis*) versus temperature. Temperatures corresponding to peaks or transitions are indicated. (**a**) *B. mori*, (**b**) *Antheraea*, (**c**) *Samia*, (**d**) *A. aliena*, *R. fugax*, and *S. jonasii*.

The cocoon materials were subjected to thermogravimetric (TG) analyses, where changes in sample mass were measured as a function of a temperature gradient from 30 to 500 °C (Fig. 3). The overall results showed that the saturniid silks had higher thermal stability compared to *B. mori*, as seen in the values for the thermal decomposition peaks (*T*_*d*_), in agreement with previous reports^34^,^35^,^36^. In most samples an initial weight loss was also seen below 100 °C, corresponding to the evaporation of adsorbed water. The exception is *R. fugax*, which produced a water evaporation peak at around 105 °C, presumably due to the dense packing of fibres in the cocoon, which could impede the loss of water molecules.

In the *B. mori samples*, no significant changes were observed after the water peak until above 200 °C, and an abrupt decrease occurred beyond 280 °C, producing a *T*_*d*_ peak at around 335 °C in the differential TG plots (DTG). Approximately 40% of the initial weight remained at the end of the run at 500 °C. For the saturniid silks, *Antheraea* and *Samia* samples showed qualitatively similar TG profiles, although the latter produced higher *T*_*d*_ values and had somewhat sharper overall features. In contrast to *B. mori*, the thermal degradation followed a two-step regime, producing a shoulder at approximately 330–340 °C in the DTG plots, followed by a more drastic decrease in mass. A similar multistep profile was reported for regenerated film derived from degummed *A. pernyi* fibres^34^. A small but distinct peak was also sometimes observed at approximately 160–170 °C in the derivative plots, presumably corresponding to the degradation of calcium oxalate crystals^37^,^38^. The wild silk samples from *A. aliena*, *R. fugax* and *S. jonasii* showed broader transitions and generally less distinct features compared to the other saturniid silks (Fig. 3).

**Figure 4:**
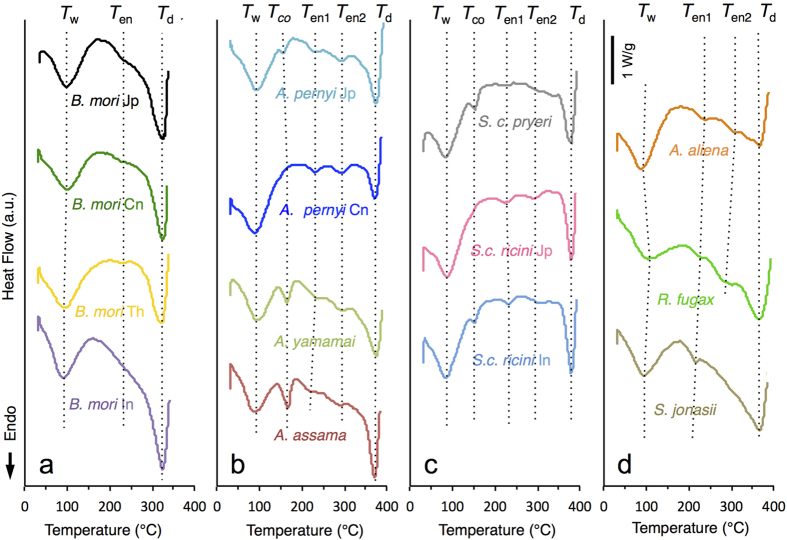
DSC measurements of the different cocoon samples. Temperatures corresponding to the peaks or transitions are indicated: *T*_*w*_ = water evaporation peak; *T*_*en*(*1,2*)_ = endothermic peaks; *T*_*co*_ = peak attributed to the degradation of calcium oxalate crystals; *T*_*d*_ = thermal degradation peak. (**a**) *B. mori*, (**b**) *Antheraea*, (**c**) *Samia*, (**d**) *A. aliena*, *R. fugax*, and *S. jonasii*.

Differential scanning calorimetry (DSC) results are shown in Fig. 4 and Table S2. Consistent with the TG data, the saturniid silks showed more highly defined profiles with a greater number of transitions compared to *B. mori*. In *B. mori* samples, aside from the *T*_*w*_ and *T*_*d*_ peaks, an indistinct endothermic peak was seen at approximately 230 °C (denoted as *T*_*en1*_). The saturniid silks, in contrast, typically produced two small, distinct endotherms (*T*_*en1*_ and *T*_*en2*_), which have been attributed to molecular motion within the amorphous or laterally ordered regions of fibroin^39^. Another small endotherm sometimes appeared between 160–165 °C, attributed to the decomposition of calcium oxalate crystals (*T*_*co*_)^37^. *A. aliena*, *R. fugax*, and *S. jonasii* displayed less defined peaks compared to the other saturniid samples, possibly reflecting a reduction in concerted molecular motions during heating. The overall results of the thermal analysis are compiled in Table S2.

### X-ray scattering and birefringence

**Figure 5:**
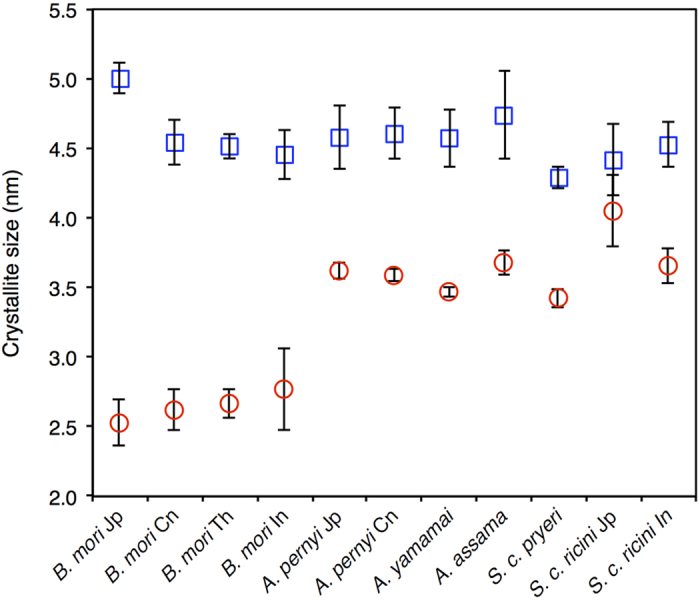
Calculated β-sheet crystallite sizes by applying Scherrer’s equation to the full width at half maximum (fwhm) of the (020) (red circle) and (210) peaks (blue square). Error bars correspond to standard errors (n = 3).

Synchrotron wide-angle X-ray scattering (WAXS) experiments were performed on the cocoon material from the different samples (Fig. S2). The *B. mori*, *Antheraea*, and *Samia* samples produced strong reflections, compared to *A. aliena*, *R. fugax*, and *S. jonasii*, which produced weakly diffracting patterns. Bragg peaks corresponding to the (020), (210), (100), and (300)/(400) reflections were identified in most cases. The 1D intensity profiles derived from azimuthal integration along the equatorial direction were fitted using Gaussian functions, and the resulting full width at half maximum (fwhm) of the (020) and (210) peaks were used to calculate crystallite sizes (Fig. S3). The results of the analysis are shown in Fig. 5, which shows average values for (020) and (210) of 2.6 nm and 4.5 nm, respectively, for the *B. mori* samples, and around 3.6 nm and 4.5 nm, respectively, for the saturniid silk samples. The degree of crystallinity of the different silk samples was also estimated from the WAXS data, as summarized in Table 1. All of the samples had crystallinity values ranging between 25–35%, suggesting a similar overall abundance of nano-crystalline structures. Birefringence was also measured to probe the degree of orientation of the crystalline units along the fibre axis (Table 1). The results showed considerably higher values for birefringence in *B. mori* compared to the saturniid silks, as in previous reports^36^,^37^, which together with the crystallinity measurements indicate an overall greater degree of order in the b-sheet along the longitudinal axes. The retardance images occasionally featured local regions of heterogeneity, suggesting certain variations in orientation of the crystalline regions caused by the natural spinning process (Fig. S4).

### Sequence analyses

**Figure 6:**
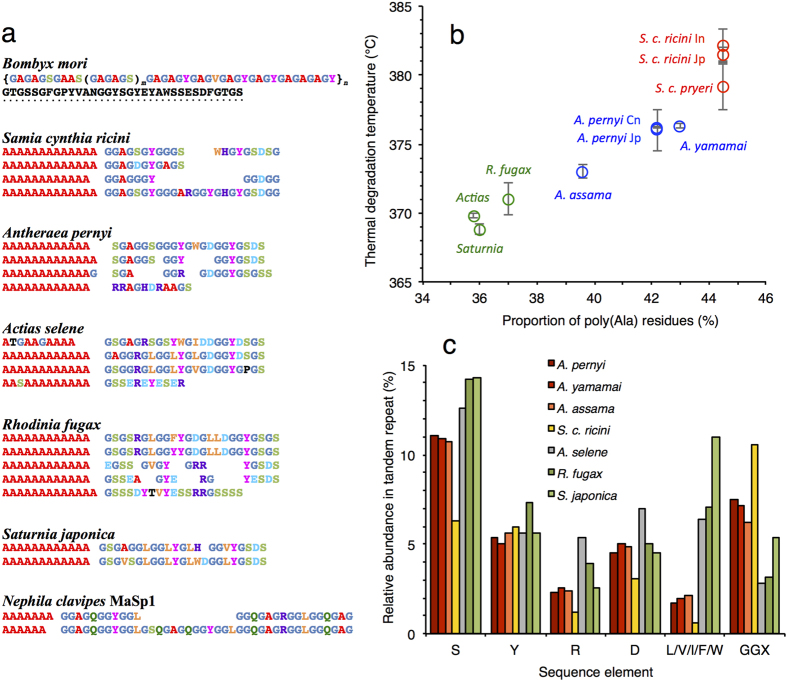
Fibroin amino acid sequence analysis. (**a**) Representative repetitive sequences of the different silk fibroin are shown: *B. mori* heavy chain (GenBank AF226688), demonstrating the hierarchical arrangement of motifs, and with the conserved spacer sequence underlined, *S. cynthia ricini* (BAQ55621), *A. pernyi* (AAC32606), *A. selene* (deduced sequence)^71^, *R. fugax* (BAG84270), *S. japonica* (BAH02016), and MaSp1 from spider dragline silk of *Nephila clavipes*^14^ (M37137). (**b**) Relative proportion of poly(Ala) residues within the saturniid fibroin tandem repeats versus the decomposition temperature (*T*_d_) of the different samples derived from TG analysis, with error bars corresponding to standard deviation values. (**c**) Average relative abundance of selected residues and motifs within the repetitive regions of the different fibroin samples.

The sequences of the repetitive fibroin domains from the different samples relevant to this study were analysed (Fig. 6). In case of *Actias aliena* and *Saturnia jonasii* only sequences from the congeneric *A. selene* and *S. japonica*, respectively, were available; these were used for the analysis on the assumption that the composition of the repetitive regions has been conserved at the genus level, as seen among *Antheraea* species^15^,^40^,^41^. It should be noted that intraspecific polymorphisms are known to occur in the silk genes, with variations in the overall length of the coding regions and in the relative arrangement of the sequence subtypes within the repetitive domains. However, based on current knowledge, the composition of tandem repeats is conserved within each species^42^,^43^,^44^,^45^.

All analysed sequences showed a preponderance of Gly, Ala, Ser, and Tyr residues; however, the organization of the internal repeats are different between the bombycoid and saturniid fibroins. The *B. mori* sequence features repetitive arrays of (GA)nGX (where X = S, Y or V), forming large blocks of varying length and arrangement that are interrupted by spacer sequences comprising ~43 residues^20^,^46^. In contrast, the saturniid repetitive domains consisted of alternating tandem repeats of poly(Ala) and non-poly(Ala) regions. The poly(Ala) blocks comprised on average 12–13 contiguous Ala residues, while the non-poly(Ala) blocks are Gly-rich, featuring various combinations of G*X* and GG*X* (*X* = typically A, S, Y, D, L or R). Interspecific variations within the non-poly(Ala) blocks were noted. The *S. c. ricini* sequence carried four subtypes bearing GG*X* motifs, while *Antheraea* sequences (*A. pernyi*, *A. yamamai*, and *A. assama*) featured three GG*X*-containing subtypes plus a shorter subtype rich in charged polar residues (*e.g.* RRAGHDRAA in *A. pernyi*). In *A. aliena*, *R. fugax*, and *S. jonasii*, the non-poly(Ala) regions contained a relatively low abundance of GG*X* motifs, but a relatively high proportion of bulky, hydrophobic residues.

Quantification of the abundance of poly(Ala) residues revealed that *S. c. ricini* contained the highest level among the saturniid repetitive sequences, at approximately 44.5% the total length of each tandem repeat, versus 43% for *A. yamamai*, 42.2% for *A. pernyi*, 39.6% for *A. assama*, 37% for *R. fugax*, 36% for *S. japonica*, and 35.8% for *A. selene*. Strikingly, plotting the values of abundance of poly(Ala) residues among the different species revealed an excellent agreement with the thermal degradation values observed from the TG experiments (Figs 6b and 3), with the highest stability corresponding to the *Samia* samples, with a *T*_*d*_ at approximately 382 °C. Conversely, the lowest abundance of poly(Ala) residues, in the *Actias*, *Rhodinia*, and *Saturnia* sequences, exhibited the lowest thermal degradation temperatures, at around 369–371 °C. These findings support a view that the poly(Ala) runs constitute the β-crystalline component of silk that imparts rigidity to the polymer structure. Interestingly, no such correlation was observed between the average lengths of the poly(Ala) stretches *per se* and degradation temperature (not shown), suggesting that the ratio of crystalline to non-crystalline fractions is the main determinant of thermal stability.

It is useful to compare the sequences of *Antheraea* with *Actias* as they are phylogenetically closely related^47^ yet produce silks with divergent material properties; the latter is particularly brittle and exhibits low extensibility, as reported here and elsewhere^9^. Both sequences harbour a similar subset of tandem repeats, although *Actias selene* carries a relatively higher proportion of residues with bulky, hydrophobic side chains (notably Leu) compared to *Antheraea*. Plotting the abundance of selected sequence elements within the repetitive regions of the different fibroins gives a clearer view of these differences (Fig. 6c). Comparing the different saturniid species, the greatest variations were seen in the relative abundance of large hydrophobic residues, namely Leu, Val, Ile, Phe, and Trp, and in the abundance of GG*X* motifs. *S. c. ricini* in particular, and the three *Antheraea* species, showed high GG*X* levels but relatively few hydrophobic residues. Conversely, the wild silks of *R. fugax*, *A. selene*, and *S. japonica* all contained much higher levels of the bulky hydrophobic residues but fewer GG*X* motifs^48^,^49^. These results suggest that the balance between the abundance of large hydrophobic residues and flexible glycine-rich motifs, both of which are situated within the fibre amorphous phase, plays an important role in determining the overall material properties of the fibres. A summary of the results from the sequence analyses is presented in Table 2.

## Discussion

The main objective of this study was to correlate the physical properties of diverse silkworm silks with the amino acid sequences of the underlying fibroins. Based on phylogeny, the silks studied belong to two main groups: the bombycoid type (represented by the *B. mori* varieties) and the saturniid type (the rest of the samples), which exhibited significant differences in the fibroin repetitive sequences at different levels of organization. The repetitive blocks in *B. mori* fibroin feature long concatenated blocks of (GA)_n_G*X* of varying lengths and arrangements, with GAGAGS repeats constituting the intermolecular β-sheets of the crystalline fraction, while the numerous Tyr residues in GAGAGY repeats are predicted to occupy semi-crystalline regions whose conformation has not yet been well resolved^50^. Due to the variability among the concatenated blocks, the sequence correspondence between laterally adjacent fibroin chains is not expected to persist over long distances, thus tending to produce heterogeneous crystalline units along the fibre. This is consistent with the large spread and general lack of consensus regarding the crystallite dimensions of *B. mori* silk^50^,^51^. The complexity inherent in the *B. mori* fibroin structure may explain the tensile test results. In particular, the gradual yielding during fibre extension (Fig. 2a) may be attributed to the non-uniform distribution of stress among the heterogeneous crystalline structures. Likewise, the relative lack of distinct features during thermal analyses could reflect a lack of concerted changes in the polymer conformations as a consequence of the heterogeneous fibre structure (Figs 3 and 4).

In contrast, the most prominent feature of the saturniid fibroin sequences is the strictly repetitive nature of the poly(Ala) and non-poly(Ala) blocks. We hypothesize that the well-defined transitions during the tensile deformation of saturniid silks reflect coordinated molecular motion that may be attributed to the high degree of modularity in the primary structures. Similarly, the sharp boundaries and well-defined endothermic peaks observed during thermal analyses (particularly in *Antheraea* and *Samia*) might be indicative of concerted effects within the fibroin chains caused by heating.

It is useful to compare the saturniid sequences with spider dragline silk (major ampullate silk), whose constituent proteins (spidroins) bear strikingly similar set of amino acid motifs, likewise arranged as alternating poly(Ala) and Gly-rich repeats (Fig. 6a). Dragline silk has been the subject of numerous investigations, and hypotheses regarding its molecular structure and function have been developed to a much greater extent compared to silkworm silks^52^,^53^. At the nanoscale, dragline silk features well-ordered crystallites (stacked β-sheets) embedded in a matrix of amorphous chains, corresponding to the ordered and disordered fractions of the silk polymers, respectively^54^,^55^. Similar to saturniid silks, the tensile deformation of dragline silk produces prominent yielding and post-yield strain hardening^11^,^56^. The yield point has been equated with a glass-to-rubber transition within the amorphous regions^57^, and post-yield strain hardening to the reconversion of the rubber states back to glass or crystalline states at higher elongation^53^. Among the silkworm sequences investigated here, *S. c. ricini* fibroin bears the closest resemblance to dragline spidroin, MaSp1, with all tandem repeat subtypes being rich in GG*X* motifs^58^; interestingly, the qualitative stress-strain profiles of the two silk types are remarkably similar^56^. It should be noted that dragline silk also includes a second spidroin component, MaSp2, whose proline-rich sequence has been linked to the extraordinary ductility of dragline silk^59^.

The three wild silks investigated (*R. fugax*, *A. aliena*, and *S. jonasii*) displayed inferior extensibility in tensile testing and relatively ill-defined transitions during thermal analyses. The corresponding fibroin sequences from these species feature a higher abundance of large hydrophobic residues within the non-poly(Ala) blocks at the expense of the GG*X* motifs compared to the other saturniids (Table 2). These bulky groups might form hydrophobic aggregates within the amorphous phase, that when combined with a low abundance of glycine residues could account for the decreased ductility (increased brittleness) in these silks. However, the biological significance of these differences in fibre properties, with respect to *in vivo* cocoon function, remains unknown.

In this study we have sought to examine the extent to which the differences in the primary structures of the fibroin molecules from different lineages of silk moths influence their material properties. Based on our results, certain relationships could be observed, for instance between the overall thermal stability of the silk fibres and the proportions of crystallite forming residues (poly-alanine) in the case of saturniid fibroins, or the correlation between brittle silk types and the preponderance of bulky hydrophobic residues within the repetitive sequences. Thus in certain respects the sequence of the constituent fibroins is a major determinant of the observable physical characteristics. However, in terms of tensile properties the overall picture is more complex. Although silk fibres from the two main lineages investigated typically follow different deformation paths that can be related to the differences in modular organization of their repetitive sequences, individual samples exhibited a wide degree of variability in their measured tensile parameters. Indeed, although the amino acid sequence of the constituent fibroin undoubtedly plays an important role in shaping material properties, other factors such as the spinning process^25^,^26^, reeling rate^60^, fibre morphology^36^,^61^, flaw distribution^30^, temperature^62^, humidity^63^,^64^ and degree of sericin binding^65^ have also been shown to modulate the mechanical properties of silk fibres. There is doubtless a subtle and complex interplay between these different intrinsic and extrinsic factors.

## Materials and Methods

### Silk samples

Fourteen types of cocoon silk were investigated: *Bombyx mori* (from four locations: Japan, China, India, and Thailand); *Antheraea pernyi* (Japan and China); *Antheraea yamamai* (Japan); *Antheraea assama* (India); *Samia cynthia pryeri* (Japan); *Samia cynthia ricini* (Japan and India); *Rhodinia fugax* (Japan); *Actias aliena* (Japan), and *Saturnia jonasii* (Japan). Samples were obtained from Shinshu University, Japan; Beihang University, China; Indian Institute of Technology, Guwahati, India; and Chulalongkorn University, Thailand. Silk fibres were unravelled from the outer surface of the unprocessed cocoons using forceps, with great care to prevent damaging the samples.

### Cross-sectional area calculation

Silk fibres were positioned on the surface of 1-mm-thick sheet of poly(methyl methacrylate) using double-sided tape on either end and covered in a thin layer of 95% cyanoacrylate and allowed to set overnight. The embedded fibres were sectioned transversely using a RM2265 microtome (Leica Microsystems, Wetzlar, Germany). Fibre cross-sections were visualized using an Olympus BX53 microscope at 500× total magnification and images were taken using Stream Essentials (Olympus, Tokyo, Japan). Cross-sectional surface areas were calculated by tracing the edge contours of the core filaments using ImageJ (NIH, Bethesda, MD, USA). Average values for surface areas were used for *A. aliena*, *R. fugax*, and *S. jonasii*.

### Scanning electron microscopy

Surface morphology of the fibres were assessed by SEM (JCM 6000, JEOL Ltd., Tokyo Japan). Samples were mounted on an aluminum stub with a conductive tape backing and sputter-coated with gold for 1 min using a Smart Coater (JEOL) prior to SEM visualization at 5 kV.

### Tensile properties of silk fibres

At least eighteen individual tensile deformation tests were performed for each silk type, with fibres were taken from three separate cocoons. The experimental setup was similar to those reported previously^32^. Each fibre was attached to a rectangular piece of cardboard with a 5 mm aperture using 95% cyanoacrylate. The tensile properties of the fibres were measured using an EZ-LX universal tester (Shimadzu, Kyoto, Japan) with a 1 N load cell, at a strain rate of 10 mm/min (0.033 s^−1^) at 25 °C and 48% relative humidity. For each tensile test, the cross-sectional area of an adjacent section of the fibre was measured. The force-displacement data were combined with surface area values to calculate tensile strength, breaking strain, and Young’s modulus. Toughness values were derived from the area under the stress-strain curves using customized software.

### Thermal analysis

Simultaneous TG and DSC were conducted in triplicate using cocoon fragments with a total mass of 3–5 mg. Samples were encapsulated in Al pans and heated under nitrogen atmosphere at a rate of 20 °C min^–1^ from 30 to 500 °C using a TGA/DSC 2 instrument (Mettler Toledo, Greifensee, Switzerland). The device was calibrated with an empty cell baseline and with indium for heat flow and temperature. The water content was calculated from the percent weight loss associated with the evaporation of bound water from the TG data^64^.

### Synchrotron WAXS measurements

Cocoon-extracted fibres were aligned in bundles and subjected to synchrotron wide-angle X-ray scattering (WAXS)^66^ at 12.4 keV at the BL45XU beam line at SPring-8 (Harima, Japan). Data collection parameters include a wavelength of 1.00 Å, beam size of 250 × 150 μm (H × V) and 10 s exposure time, at 25 °C and 40% relative humidity. Diffraction patterns were recorded using Pilatus 2 M (DECTRIS Ltd., Switzerland) with a sample-to-detector distance of 179.6 mm. The module gaps of the detector by offset measurement was complemented. The 2D diffraction patterns were converted into 1D profiles using Fit2D^67^, with corrections made for background scattering and detector geometry. The 1D intensity profiles derived from azimuthal integration along the equatorial direction were fitted with Gaussians corresponding to the main Bragg peaks and the amorphous halo and sericin contribution^66^, plus a constant for the residual background scattering. The lower size limit of the crystallite sizes were estimated using Scherrer’s equation, *L* = (0.9 *λ*)/(*B* cos *θ*), with *B* corresponding to the full width at half maximum (fwhm) of the Gaussians fitted to the (020) and (210) reflections^68^,^69^. Crystallinity values of the cocoon silks were calculated from the 1D profiles^68^,^70^. Each data set was separated into crystalline and amorphous scattering components by curve fitting using Gaussian functions. The ratio of the total area of the separated crystalline scattering components to that of the crystalline and amorphous scattering components was used to determine the crystallinity of the samples.

### Birefringence

The degrees of alignment of crystalline elements within the silk fibres were assessed by measuring birefringence, calculated as the retardance per unit of thickness. Retardance measurements were taken using WPA-View software v.1.08 (Photonic Lattice, Inc.) on an Olympus BX53 polarized light microscope at 500× total magnification. Retardance was converted into birefringence by dividing by the thickness of the fibres in nm.

### Sequence analysis

Amino acid sequences of the fibroins used for the sequence analysis were retrieved from GenBank: *Bombyx mori* (GenBank accession code AF226688; AAB31861), *Samia cynthia ricini* (BAQ55621), *A. pernyi* (AAC32606), *A. yamamai* (BAJ11925; AB542805), *A. assama* (KJ862544), *Rhodinia fugax* (BAG84270), *Saturnia japonica* (BAH02016), and *Actias selene* (ADA59934). Additional sequences were obtained from *de novo* assembled transcriptome data for *A. assama* and *A. selene*^71^). Intragenic tandem repeats of saturniid fibroins were aligned using Geneious R9 (Biomatters Ltd., Auckland, NZ) and scored for the frequency of conserved amino acid residues and motifs. The final tandem repeat flanking the C-terminus was not included in the scoring as this segment is typically divergent from the rest of the repetitive regions.

### Statistical analysis

Tukey’s HSD (Honestly Significant Difference) test was used in conjunction with one-way analysis of variance (ANOVA) for single step multiple comparisons to analyse the results of the tensile deformation tests, using IBM SPSS Statistics for Macintosh v. 22.

## Additional Information

**How to cite this article**: Malay, A. D. *et al*. Relationships between physical properties and sequence in silkworm silks. *Sci. Rep.* **6**, 27573; doi: 10.1038/srep27573 (2016).

## Supplementary Material

Supplementary Information

## Figures and Tables

**Table 1:** Calculated values for crystallinity and birefringence.

Sample	Crystallinity (%)	Birefringence
*B. mori* Jp	33.7	0.045
*B. mori* Cn	30.2	0.069
*B. mori* In	30.5	0.043
*B. mori* Th	34.3	0.037
*A. pernyi* Jp	26.4	0.014
*A. pernyi* Cn	30.9	0.007
*A. yamamai*	32.5	0.016
*A. assama*	34.7	0.02
*S. c. pryeri*	31.1	0.032
*S. c. ricini* Jp	31.3	0.026
*S. c. ricini* In	25.8	0.033
*A. aliena*	25.7	0.02
*R. fugax*	28.7	0.009
*S. jonasii*	30.3	0.008

**Table 2:** Comparison of sequence features in the repetitive regions of the different silks.

Genus	(GA)_n_GX	Extended linker	poly(Ala)	GGX	L/V/I/F/W	non-GGX repeat
*Bombyx*	✓	✓	x	x	*NA*	*NA*
*Samia*	x	x	+ + +	+ + +	+	x
*Antheraea*	x	x	+ +	+ +	+ +	✓
*Actias*	x	x	+	+	+ + +	✓
*Rhodinia*	x	x	+	+	+ + +	✓
*Saturnia*	x	x	+	+	+ + + +	x
