# Supplementary material for: Relationships between physical properties and sequence in silkworm silks
Source: Sci Rep. 2016 Jun 9;6:27573. doi: 10.1038/srep27573 (PMC4899792; doi:10.1038/srep27573)
Supplement: Supplementary Information [file srep27573-s1.pdf]

Supplementary Information

Relationships between physical properties and sequences in silkworm silks

Ali D. Malay, Ryota Sato, Kenjiro Yazawa, Hiroe Watanabe, Nao Ifuku, Hiroyasu Masunaga, Takaaki Hikima, Juan Guan, Biman B. Mandal, Siriporn Damrongsakkul, Keiji Numata

- Fig S1-S4
- Table S1-S2

Supplementary Figure S1. Compilation of representative results from tensile deformation tests of the 14 silk samples used in the study.

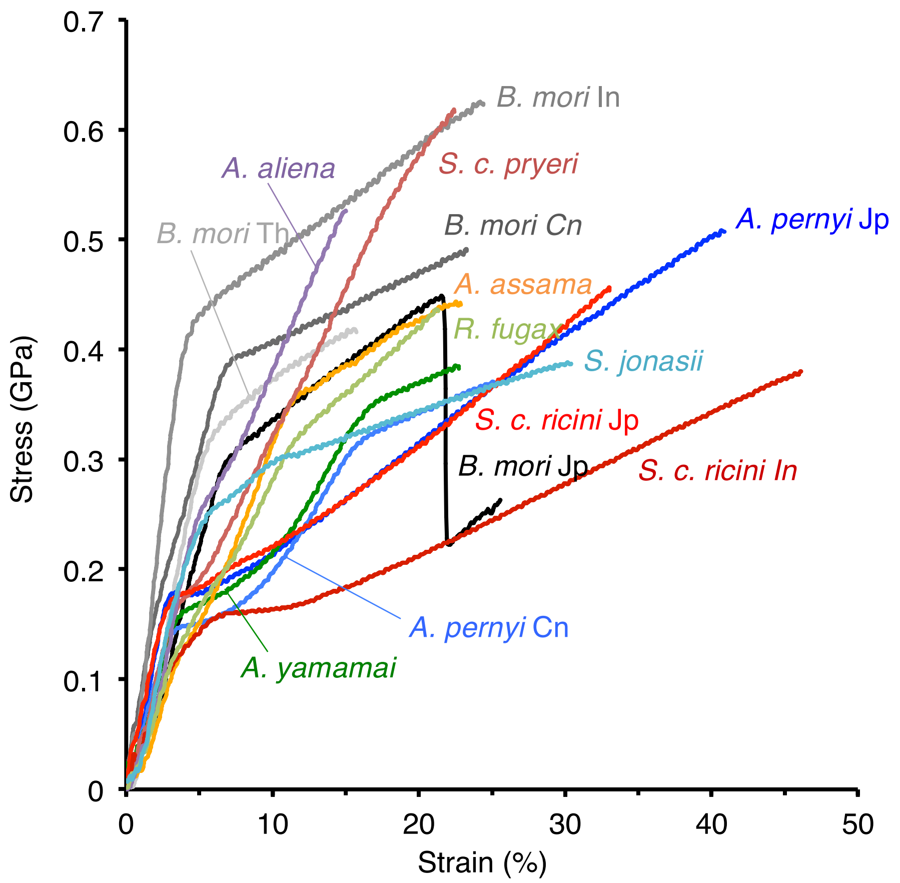

Supplementary Figure S2. WAXS reflection patterns of the different silks. (a) *B. mori* Jp; (b) *B. mori* Cn; (c) *B. mori* Th; (d) *B. mori* In; (e) *A. pernyi* Jp; (f) *A. pernyi* Cn; (g) *A. yamamai*; (h) *A. assama*; (i) *S. c. ricini* Jp; (j) *S. c. ricini* In; (k) *S. c. pryeri*; (l) *A.aliena;* (m) *R. fugax*; (n) *S. jonasii*.

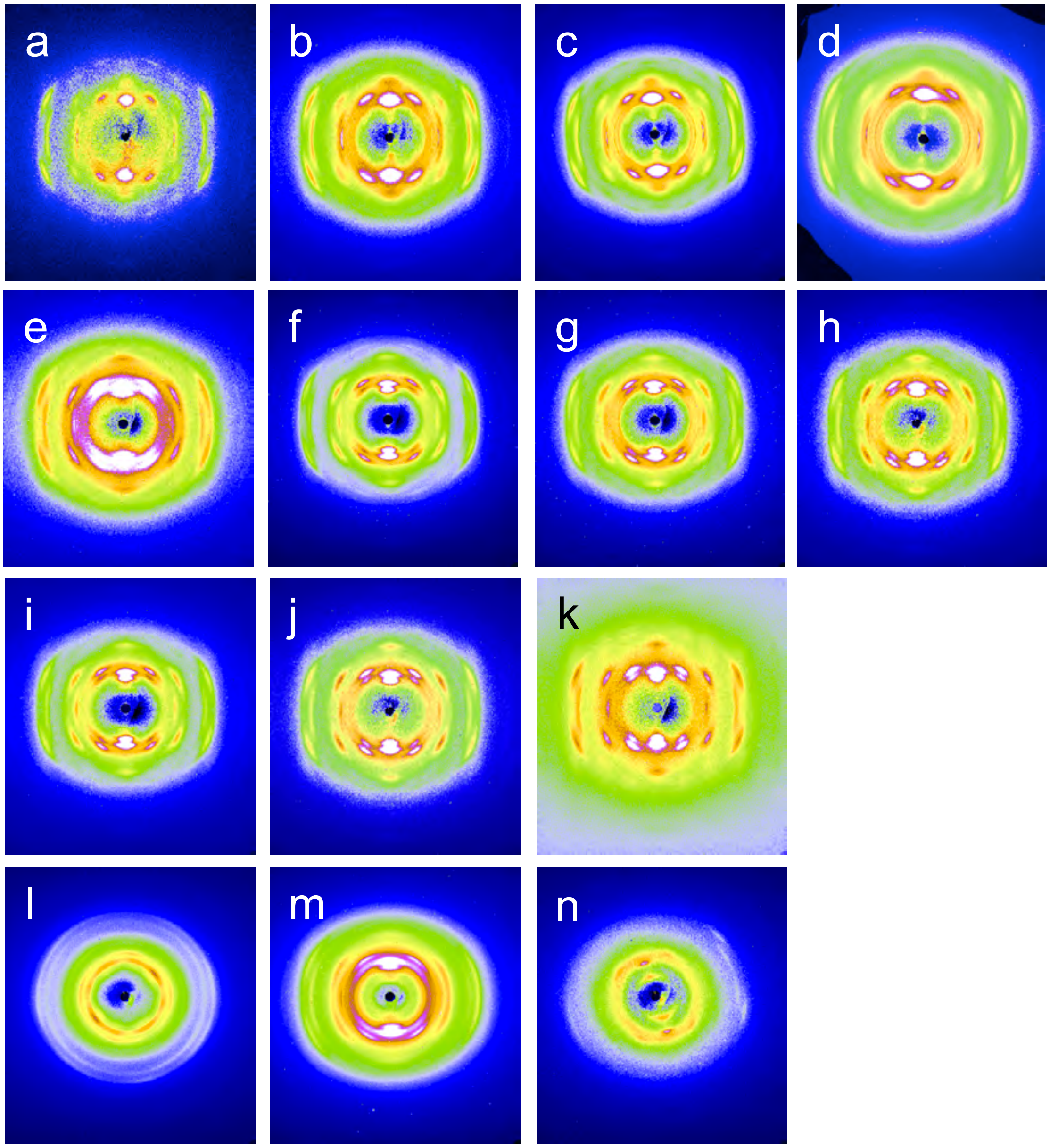

Supplementary Figure S3. (a, b) 2D diffraction images indicating main reflections. (b, c) 1D profiles of equatorial scans of the WAXS data showing Gaussian functions fitted to the main peaks and amorphous halo. (d, e) 1D profiles of meridional scans showing attempted fit of Gaussians to the data. Panels to the *left* (a, c, e) correspond to *B. mori* Cn and figures to the *right* (b, d, f) correspond to *S. c. ricin*i In.

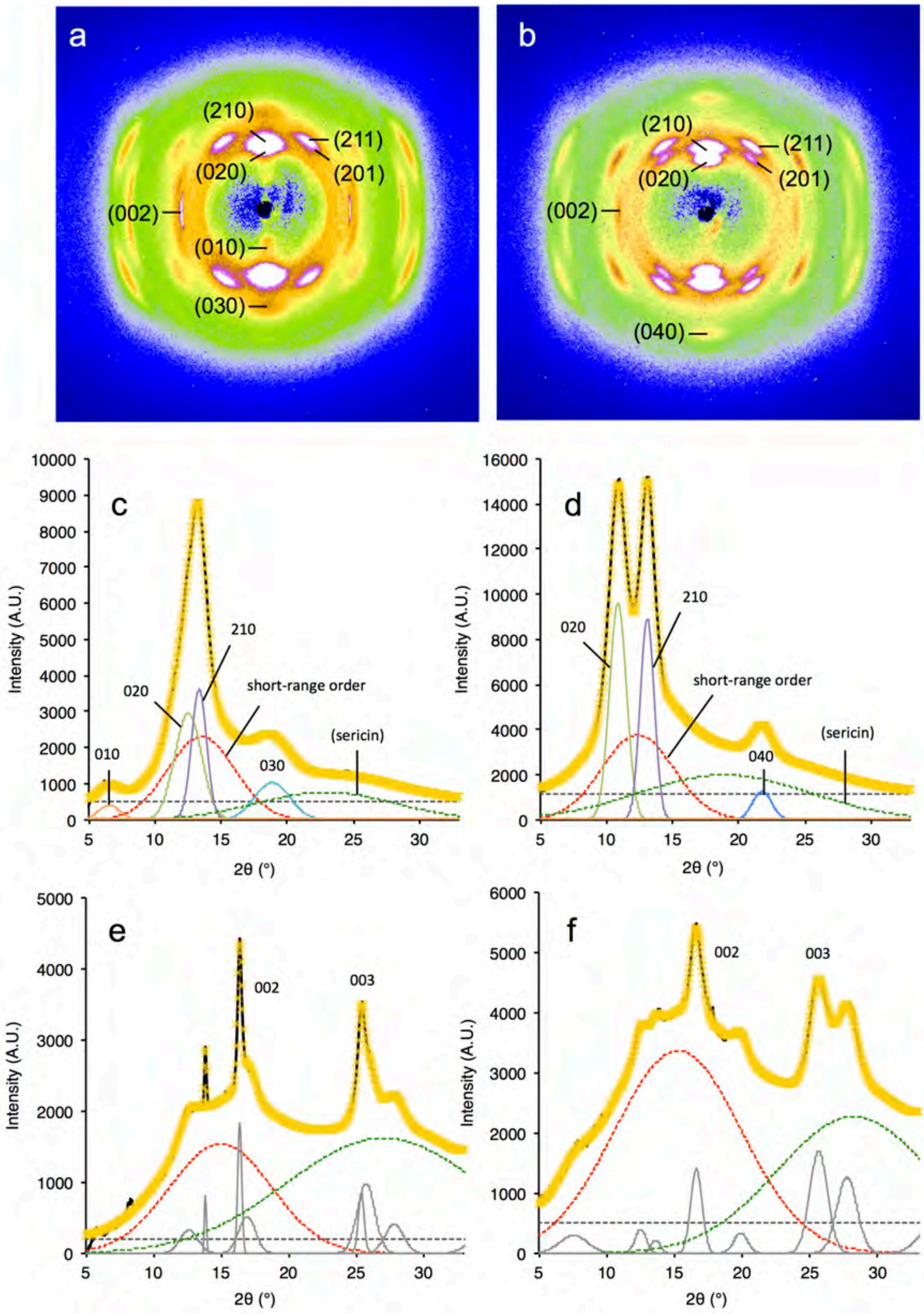

Supplementary Figure S4. Retardance images used for birefringence calculations. (a) *B. mori* Jp; (b) *B. mori* Cn; (c) *B. mori* Th; (d) *B. mori* In; (e) *A. pernyi* Jp; (f) *A. pernyi* Cn; (g) *A. yamamai*; (h) *A. assama*; (i) *S. c. ricini* Jp; (j) *S. c. ricini* In; (k) *S. c. pryeri*; (l) *A. aliena*; (m) *R. fugax*; (n) *S. jonasii*.

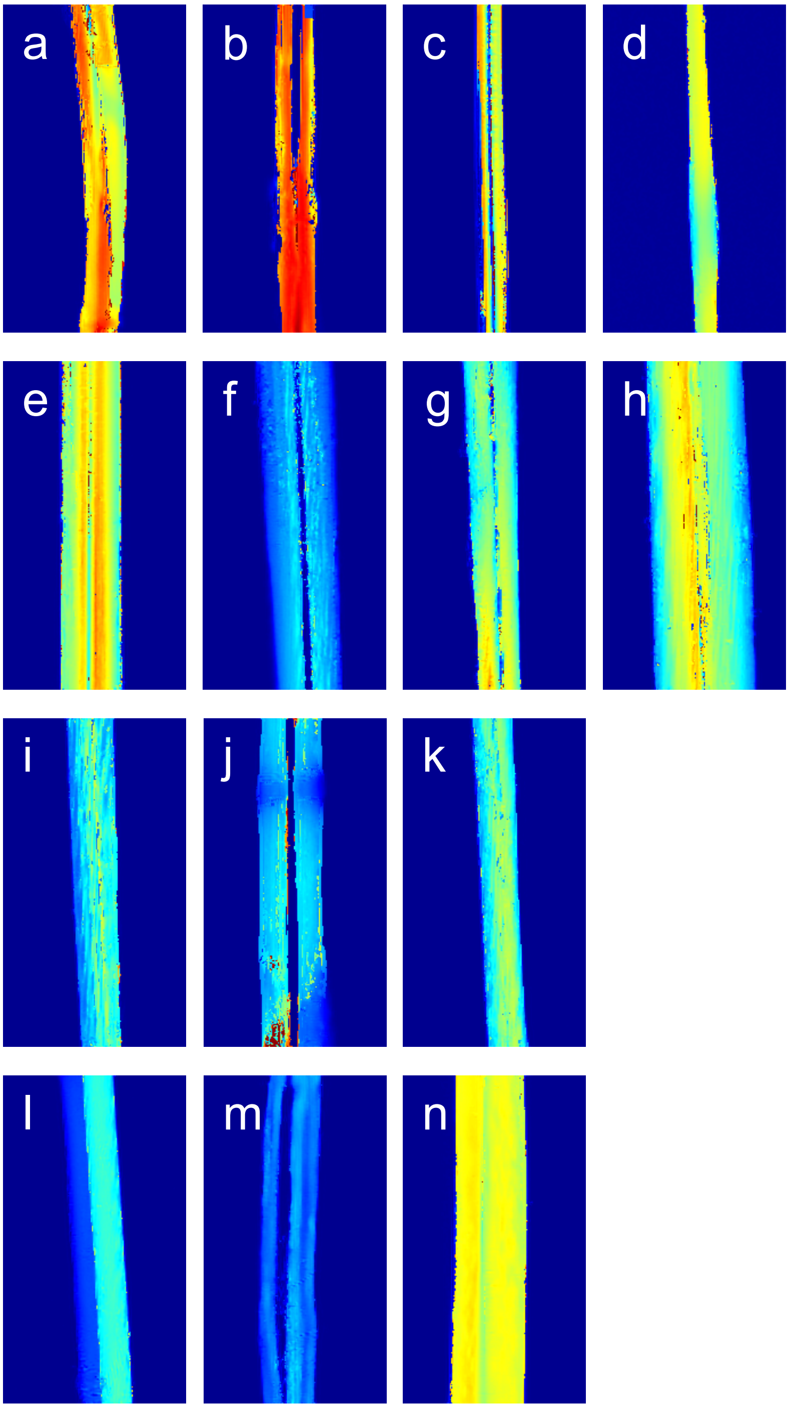

Supplementary Table 1. Summary of results from tensile deformation tests on individual silk fibres, showing standard deviation values.

Sample	Tensile Strength (GPa)	Extensibility (%)	Young's Modulus (GPa)	Toughness (GJ m^{-3})
B. mori Jp	0.40 ± 0.11^{ab}	26.8 ± 5.8^{abcd}	5.13 ± 1.6^{abc}	0.071 ± 0.028^{abc}
B. mori Cn	0.46 ± 0.08^{abc}	24.1 ± 7.7^{abcd}	7.23 ± 1.68^{cd}	0.081 ± 0.034^{bc}
B. mori Th	0.55 ± 0.11^{c}	23.2 ± 7.9^{abcd}	6.66 ± 1.06^{bcd}	0.091 ± 0.042^{bc}
B. mori In	0.57 ± 0.12^{c}	24.5 ± 10.1^{abcd}	8.61 ± 2.19^{d}	0.103 ± 0.057^{c}
A. pernyi Jp	0.34 ± 0.08^{a}	26.7 ± 8.4^{abcd}	4.72 ± 1.73^{ab}	0.060 ± 0.020^{abc}
A. pernyi Cn	0.43 ± 0.08^{abc}	29.6 ± 10.0^{bcd}	4.76 ± 1.09^{ab}	0.079 ± 0.027^{abc}
A. yamamai	0.39 ± 0.07^{ab}	35.6 ± 13.7^{d}	4.58 ± 0.58^{ab}	0.092 ± 0.043^{bc}
A. assama	0.36 ± 0.10^{a}	29.2 ± 10.7^{bcd}	4.31 ± 1.15^{a}	0.068 ± 0.031^{abc}
S. c. pryeri	0.53 ± 0.15^{bc}	24.1 ± 5.2^{abcd}	4.71 ± 1.26^{ab}	0.078 ± 0.023^{abc}
S. c. ricini Jp	0.38 ± 0.12^{a}	33.9 ± 11.4^{cd}	4.61 ± 2.01^{ab}	0.078 ± 0.038^{abc}
S. c. ricini In	0.47 ± 0.11^{abc}	29.4 ± 8.0^{bcd}	4.69 ± 1.26^{ab}	0.086 ± 0.030^{bc}
R. fugax	0.43 ± 0.01^{abc}	22.3 ± 1.2^{abc}	4.09 ± 0.28^{a}	0.058 ± 0.003^{abc}
A. aliena	0.39 ± 0.14^{ab}	14.9 ± 3.7^{a}	4.69 ± 1.98^{ab}	0.030 ± 0.010^{a}
S. jonasii	0.37 ± 0.10^{a}	19.6 ± 13.0^{ab}	7.10 ± 2.60^{cd}	0.050 ± 0.040^{ab}

Values in the same column with different superscript letters are significantly different at $p < 0.08$.

Supplementary Table 2. Summary of results from combined TG and DSC experiments.

Sample	Water content (%)	T_w	T_{en1}	T_{en2}	T_d
B. mori Jp	6.68 ± 0.79	96.05 ± 3.35	239.91 ± 2.72		335.36 ± 0.45
B. mori Cn	5.37 ± 0.41	93.63 ± 1.89	235.17 ± 5.70		338.04 ± 0.85
B. mori Th	5.96 ± 0.14	92.08 ± 0.93	224.36 ± 7.10		327.07 ± 0.18
B. mori In	8.11 ± 0.52	87.51 ± 0.05	231.97 ± 3.56		328.42 ± 0.62
A. pernyi Jp	7.65 ± 0.30	92.71 ± 0.56	230.58 ± 1.76	295.44 ± 1.27	375.96 ± 1.50
A. pernyi Cn	7.43 ± 0.69	91.67 ± 1.44	230.72 ± 0.59	296.50 ± 3.29	376.16 ± 0.09
A. yamamai	7.91 ± 0.20	97.37 ± 2.01	229.69 ± 0.61	292.99 ± 4.99	376.31 ± 0.18
A. assama	7.62 ± 0.58	88.61 ± 2.06	220.41 ± 2.68	287.04 ± 3.03	372.99 ± 0.47
S. c. pryeri	7.55 ± 0.20	87.78 ± 0.70	227.82 ± 3.24	290.48 ± 10.06	379.19 ± 1.66
S. c. ricini Jp	7.34 ± 0.24	87.48 ± 0.18	230.54 ± 6.75	300.80 ± 9.39	381.51 ± 0.48
S. c. ricini In	8.35 ± 0.53	87.97 ± 1.18	231.28 ± 0.91	299.18 ± 0.24	382.14 ± 1.22
R. fugax	7.12 ± 0.21	105.26 ± 2.72	224.16 ± 0.53	291.01 ± 3.03	371.04 ± 1.17
A. aliena	7.80 ± 0.46	89.15 ± 2.24	233.94 ± 0.77	307.35 ± 3.74	369.79 ± 0.20
S. jonasii	7.92 ± 0.32	94.07 ± 3.96	215.27 ± 1.00		368.78 ± 0.35
